# Influence of sound vibrations on plant holobionts: physiological pathways linking root function and rhizospheric microbial interactions

**DOI:** 10.1080/15592324.2026.2659424

**Published:** 2026-04-15

**Authors:** Hafiza Komal Naeem, Diego Comparini, Bruno Bighignoli, Giulia Mozzo, Felipe Yamashita, Luciana Renna, Giovanni Stefano, Stefano Mancuso, Elisa Masi

**Affiliations:** a Department of Agriculture, Food, Environment and Forestry (DAGRI), University of Florence, Florence, Italy; b Sant'Anna School of Advanced Studies, Pisa, Italy; c Biosciences, Biotechnology and Environemnt (DBBA), University of Bari, Bari, Italy; d Department of Biology, University of Florence, Florence, Italy

**Keywords:** Plant holobiont, root exudation, acoustic vibration, stress resilience, sustainable agriculture, plant bioacoustics

## Abstract

Climate change increasingly threatens plant productivity and ecosystem stability, highlighting the need for sustainable strategies that enhance plant resilience. The plant holobiont—comprising the plant and its associated rhizospheric microbiota—has emerged as a key functional unit governing plant performance under environmental stress. Among emerging non-invasive approaches, sound and vibration stimuli have been reported to influence plant growth, stress responses, and microbial activity; however, the physiological mechanisms underlying these effects remain poorly defined. This review synthesizes current evidence on sound-induced plant and microbial responses within a holobiont framework and advances a physiology-driven conceptual model linking acoustic stimuli to root function and rhizospheric processes. We propose that sound vibrations act primarily as mechanical cues perceived by plant tissues through mechanotransduction pathways, triggering calcium and hormonal signaling that modulate root architecture, metabolism, and exudation patterns. These root-level physiological changes are hypothesized to indirectly shape rhizospheric microbial community assembly and function, thereby influencing nutrient acquisition, stress tolerance, and agronomic performance. By explicitly connecting sound perception, root functional traits, and plant-mediated microbial responses, this review moves beyond a descriptive synthesis and provides a mechanistic framework to guide future experimental research. Understanding these pathways may support the development of sound-based strategies as low-impact tools for improving plant–soil–microbe interactions in sustainable agriculture.

## Introduction

Agricultural science and technology are advancing rapidly, with new developments emerging each year. However, this progress has also led to environmental challenges such as pollution and ecological degradation. While chemical agriculture, through the use of fertilizers and pesticides, has enhanced crop production and yield, it has also posed serious risks to human health. Therefore, adopting sustainable agricultural practices has become essential.^1^ In the modern era, the agricultural sector is increasingly embracing ecological farming practices as a sustainable alternative to chemical-based methods. Ecological agriculture integrates modern techniques with traditional approaches to achieve greater economic, environmental, and social benefits.^2^ Nowadays, sound vibrations are being used to enhance plant growth,^3^ metabolism,^4^ stress-related gene expression,^5^ the production of secondary metabolites^6^ and resistance to diseases.^7^ Throughout this review, the term ‘sound’ is used broadly to include airborne acoustic waves and vibration-based mechanical stimuli.

Sound waves are a category of pressure waves produced by vibrating objects through any medium, including solid, liquid, and gas. Sounds can be characterized by their intensity (dB) and frequency (Hz). One cycle of sound consists of one oscillation. Oscillation frequency can be calculated in Hertz (Hz) units. As a result, 1 Hertz (1 Hz) is equal to 1 cycle per second. It can be expressed as 1 Hz = 1 cycle s^−1^.

A sound measurement scale is necessary because the change in acoustic pressure results in massive variation. Logarithms are used in these scale measurements of sounds to condense the massive variations. In the acoustic field, the sound pressure scale has been defined already. One decibel (1/10 bel) can be defined as the change in the amount of energy on every 10 factors. In common, a decibel (dB) is frequently used as a sound unit instead of 1 bel because it is too huge to be used (1 × 10^−1^ of a bel).

Moreover, the Logarithm of two intensities can be used to express the sound intensity level (*SIL*).

SIL(dB)=10log(I/Iref).

In the equation, I_*ref*_ represents the reference intensity, and *I* denotes the intensity of the sound. In the air, the intensity of sound decreases as 1/*r*^2^, where r is the distance from the sound source.^8^ In general, the speed of sound is directly related to the elasticity of the medium and inversely related to its density. Additionally, the intensity of sound also depends on the medium through which the sound waves are traveling.^9^ Audible sounds range from 20 Hz to 20 kHz, which is the frequency range that the human ear can detect. Sounds below 20 Hz are known as infrasound, while those above 20 kHz are referred to as ultrasound.^9^ However, sound is not just vibrational energy; it is also pressure generated by vibration waves that travel through a suitable medium in the form of compression and rarefaction.^9^ Infrasound and ultrasound have been utilized as tools in the medical industry for the diagnosis and treatment of various diseases.^10^^,^^11^

Sound is a fundamental medium used for both interkingdom and intrakingdom communication. Plants are known to communicate through volatile organic compounds (VOCs), such as fatty acid-derived molecules like green leaf volatiles (GLVs), benzenoids, terpenoids, and phenylpropanoids.^12^ Another form of plant communication occurs through the microbial, insect, and fungal networks associated with the roots of plants. These interactions facilitate the exchange of signals and resources, enhancing plant health and defense mechanisms.^13^ It is therefore plausible that plants may also use sound as a means of communication, despite being sessile organisms and lacking any sound-perceiving organs.

Recent studies have tried to understand how plants react to sound waves.^14^ It has been reported that the physiological changes occur as a response to sound stimuli in plants. The immunity of *Arabidopsis thaliana* is enhanced through the production of glucosinolates (GSs) and anthocyanin defensive compounds in response to the sound vibrations mimicking insect chewing.^15^ Additionally, *Arabidopsis thaliana* also demonstrated a response by producing salicylic acid and jasmonic acid when exposed to 500 Hz sound vibrations.^16^

Beyond influencing plant physiology, sound waves may also impact the rhizospheric microbial community, a highly diverse niche inhabited by a variety of microorganisms that interact with plants and contribute to numerous regulatory mechanisms.^17^ The rhizosphere, the region surrounding plant roots, hosts a significantly higher microbial community compared to non-rhizosphere areas.^18^^,^^19^ The interactions between plants and the rhizospheric microbiome are diverse, encompassing both positive and negative symbiotic relationships, including mutualism, parasitism, and commensalism.^20^ However, only a few studies have explored the impact of sound on rhizospheric microbes. For instance, one experiment demonstrated that exposing corn plants to sound for seven days led to a 23.08% increase in the population of phosphate-solubilizing bacteria in the rhizosphere. This sound treatment not only enhanced the microbial population but also increased the diversity and activity of these beneficial microbes.^21^ In another study by Munar et al., phosphate-solubilizing bacteria were isolated from sound treated rhizosphere. The identified bacterial species included *Burkholderia contaminans*, *B. cepacia*, *B. latens*, and other *Burkholderia* species. Additionally, phosphate-solubilizing fungi were also isolated and identified from the sound treated rhizosphere, including *Talaromyces muroii* and other *Talaromyces* species.^22^

Plant growth-promoting rhizobacteria and mycorrhizal associations enhance plant growth, improve nutrient availability, and strengthen defense mechanisms against biotic and abiotic stresses, resulting in a positive impact on plant health.^23‐25^ In contrast, negative interactions between plants and microbes lead to pathogenic relationships, resulting in disease development.^26^^,^^27^ Chemical and specialized molecular signaling are responsible for making a relation with the plant that either cooperative, competitive, or any other behavior. It makes the plant microbial interaction or the microbe microbial interaction.

To enhance plant productivity through the natural microbiota, there is a growing need to adopt sustainable, cost-effective, and eco-friendly agricultural practices. This review focuses on an emerging area of interest: the impact of sound waves on plant growth and their interaction with the holobionts. Holobionts are the complex ecological unit comprising the plant and its associated microbial communities. Special attention is given to the microbiota, highlighting that sound stimulation can positively influence microbial growth, diversity, and functionality, thereby enhancing nutrient availability and improving overall plant productivity. Existing research suggests that acoustic signals not only affect plant morpho-physiological but also modulate the composition and activity of root-associated microbes, influencing key plant–microbe symbioses. Accordingly, the objective of this review is to shed light on the role of sound in shaping the root microbiome and mediating plant–microbe interactions, particularly within the rhizosphere. This area remains relatively unexplored, highlighting a significant knowledge gap and the need for further targeted investigations. The literature discussed was selected to represent key experimental and conceptual advances rather than to provide an exhaustive systematic survey; it was sourced from established academic databases, including Google Scholar, Scopus, Web of Science, and other relevant scientific repositories.

## Plant bioacoustics: molecular mechanisms and functional responses

Application of sound at different frequencies, pressure levels, duration, and repetition of exposure periods has been proven to influence plant growth, development, and germination^28^ Taken together, these studies belong to the field of Plant Acoustics. Whether or not sound perception and emission are used in plant communication is a fascinating field of research. While not yet fully established, it is conceivable that plant acoustic interactions operate through a sophisticated source-receiver model. This would imply not only the detection of sound waves but also a level of signal processing that enables a coherent biological response, representing a fascinating frontier in plant communication studies.

Supporting this theoretical framework, recent evidence suggests that plants actively use ultrasonic signals to manage their water loss. Recent discovery with the potential to revolutionize smart agriculture and water conservation. Ultimately, decoding how plants talk through sound transcends basic research. It offers a new paradigm for environmental protection and paves the way for a more sustainable future.^29^

Table 1 summarizes experimental evidence for sound-induced plant responses and interprets these observations in terms of putative physiological processes and agronomic relevance, providing the empirical basis for the conceptual framework proposed in Table 3.

**Table 1.** Plant responses to sound stimulation: experimental evidence, putative physiological processes, and agronomic relevance.

Plant species	Sound treatment	Observed response	Putative physiological process	Agronomic/horticultural trait	References
*Arabidopsis thaliana*	100 dB white noise; 100 Hz and 100 + 9 kHz	Improved growth, altered phytohormone levels, drought tolerance	Ca^2+^ signaling, auxin/cytokinin rebalancing, ABA reduction	Root development, drought resilience	[30,31]
*Oryza sativa* (rice)	100 dB sound	Enhanced relative water content, photosynthesis, drought tolerance	Mechanotransduction, ABA-mediated stress signaling	Improved water-use efficiency	[32]
*Capsicum* spp. (pepper)	500 Hz	Improved germination and growth	Hormonal priming, cell expansion	Early vigor and establishment	[33]
*Ipomoea aquatica* (water spinach)	4000 Hz, 30 min/d	Increased stomatal index and photosynthesis	Stomatal regulation, mechanosensitive signaling	Yield and biomass increase	[34]
*Sansevieria trifasciata*	600–1600 Hz	Enhanced nutrient uptake	Root membrane permeability, ion transport	Nutrient-use efficiency	[35]
*Dendrobium candidum*	1000 Hz, 100 dB	Enhanced antioxidant activity and defense	ROS–Ca^2+^ signaling, antioxidant induction	Stress tolerance and plant quality	[36]
*Dendranthema morifolium* (callus)	1000 Hz	Increased soluble proteins and sugars	Metabolic activation, cytoskeletal reorganization	Growth and biomass accumulation	[37]
*Gossypium* spp. (cotton)	Audible sound waves	Increased leaf size, plant height, productivity	Hormonal modulation, enhanced photosynthesis	Yield improvement	[38]
*Brassica juncea*	4000–5000 Hz	Improved growth performance	Cell elongation and metabolic activation	Biomass and productivity	[39]
*Nicotiana tabacum*	1000 Hz	Altered cytosolic Ca^2+^ and membrane integrity	Ca^2+^ influx, membrane mechanotransduction	Stress acclimation	[40]
*Chrysanthemum*	1000 Hz) once for 3, 6, 9, 12, and 15 d	Accelerated the synthesis of RNA and soluble protein	The content of DNA but accelerated the synthesis of RNA and soluble protein	Switch on stress-induced genes	[41]
*Chinese broccoli (Brassica alboglabra)*	Frequencies ranged from 3–5 kHz, 7–9 kHz, and 11–13 kHz	Significant effect on plant wet weight, plant length, and stomata openings	Stomatal regulation	Productivity and quality of Kailaan plants	[42]

Note: direct comparison among studies is limited by substantial differences in sound intensity, exposure duration, and experimental setup.

One topic of study related to plant responses to sound involves trichomes, hair-like structures present on plant epidermis. It is considered that they can act as the antennae that receive the sound stimuli and trigger further downstream signaling responses.^43^ At the same time, it is a fact that trichomes are very varying structure and having the diversity in the number size and density across the plant kingdom so by considering these elements it is sure that trichomes are not indispensable for airborne sound perception. Rather, trichomes are more likely to be involved in the mechanical transmission or modulation of acoustic and vibrational stimuli from the air, the soil, or the plant surface itself.^44^ Similarly, also the root system responds to the mechanical stimuli, for example converting sound waves in the biochemical signals. That activates the further process in the cell that leads to the change in growth, development, biochemical and biochemical changes. However, it is not clear yet how the root system perceives the sound, and the topic is under debate.^31^^,^^45^

However, sound vibrations can trigger ion fluxes in plant cells by creating tension in the cell membrane, which activates mechanosensitive ion channels. The plasma membrane is flexible and contains specialized transmembrane structures that sense mechanical stimuli.^46^ Channels such as MSL and non-selective channels regulate H⁺, Na⁺, and Cl⁻ fluxes.^47^ While two-pore potassium channels (TPKs) mediate K⁺ movement in response to sound.^48^ Calcium ions (Ca^2+^) are particularly important, and their transport is controlled by several channels, including VICCs,^49^ MSL,^47^ PIEZO,^50^ GLRs,^51^ DEK1,^52^ MCA,^53^ OSCA,^54^ and CNGCs.^55^

Small molecules like oligosaccharides, attached to the cell wall, can interact with mechanoreceptors in the membrane to further influence ion channel activity, altering the electrical and chemical gradients of the cell.^56^ The activation of these channels not only regulates ion fluxes but also contributes to plant defense, structural integrity, osmotic stress tolerance, and maintenance of cell size and shape.^48^^,^^57^ Activation of mechanosensitive channels generates Ca^2+^ waves, which are further modulated by calmodulin, calcium-dependent protein kinases, CBL-interacting protein kinases, and calcineurin B-like proteins.^16^ These sensors, located in organelles like mitochondria, Golgi apparatus, and vacuoles, allow plants to perceive sound vibrations and translate them into morpho-physiological, biochemical, and molecular responses.^58^^,^^59^

Reactive nitrogen species (RNS), particularly nitric oxide (NO), interact with reactive oxygen species (ROS) and H₂S to modulate physiological and developmental processes, including flowering, senescence, and root growth.^60^^,^^61^ Although the effect of sound on NO production has not been fully explored, NO can influence Ca^2+^ channel activity and protein post-translational modifications.

Sound stimuli also increase ROS production, which works reciprocally with Ca^2+^ to trigger cellular responses, including epigenetic modifications and gene expression changes.^16^^,^^62^^,^^63^ The increased ROS is balanced by antioxidant enzymes such as superoxide dismutase (SOD), catalase (CAT), peroxidases (POD), and antioxidants like ascorbic acid (AA), tocopherol, and glutathione (GSH) Protein kinases, particularly proline-rich extension-like receptor kinases, play a central role in signal transduction by phosphorylating proteins in response to mechanical stimuli.^64^ Other kinases, such as lectin receptor-like kinases, leucine-rich repeat receptor-like kinases, ANXUR1/2, THESEUS (THE1), FERONIA (FER), and wall-associated kinases (WAKs), can respond to sound vibrations and are involved in processes like actin filament growth.^65^^,^^66^ Overall, it is proposed that mechanosensitive ion channels and their downstream signaling pathways could enable plants to sense and respond to sound vibrations. This mechanism potentially links physical stimuli from microbes or the environment to physiological and molecular adaptations, though further empirical evidence is needed to fully confirm these pathways.

However, there is still a less definitive understanding of how plants respond to sound or how these responses are reflected at the molecular level. Extensive future research is needed to explore how sound stimuli influence molecular activity and, in turn, affect plant growth. Various factors must be taken into account, such as the type of sound (ultrasound or infrasound), the experimental setup, the physiological and molecular responses of the plants, and the specific plant species being studied. Therefore, it is strongly recommended that these aspects be carefully considered and further investigated in future research.

## Impact of acoustic stimuli on microbial communities and plant-microbe interactions

**Figure 1.**
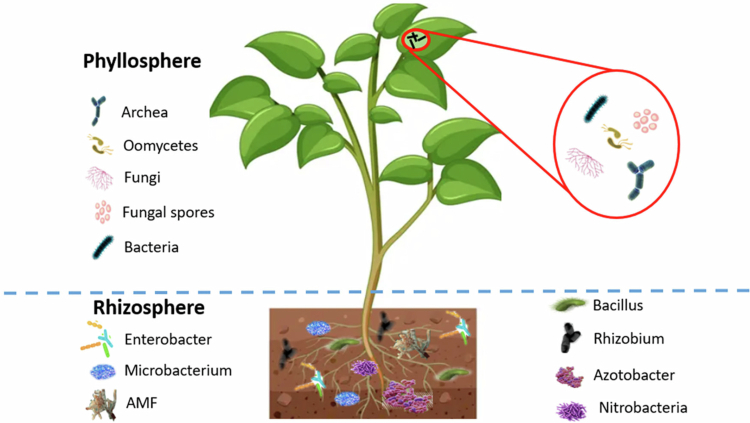
Illustrating the higher microbial density, diversity, and richness in the rhizosphere compared to the phyllosphere, with dominant genera including *Enterobacter*, *Bacillus*, *Rhizobium*, *Azotobacter*, *Nitrobacter*, *Fusarium*, *Streptomyces*, and *Pseudomonas* (Data extracted from.^67‐69^

Many studies have revealed that due to the rhizospheric effect, there is more microbial community density, variety, and richness in the rhizospheric soil than in ordinary soil.^67^ Figure 1 illustrates the microbial richness in the rhizosphere as compared to the other soil and phyllosphere. Microbial species such as *Enterobacter*, *Microbacterium*, *Bacillus*, *Rhizobium*, *Azotobacter*, and *Nitrobacter* are more abundantly present in the rhizosphere. In contrast, the phyllosphere hosts relatively fewer microbial communities. Therefore, soil-associated microbes have a greater impact on plant responses compared to microbes inhabiting the phyllosphere. *Fusarium*, *Streptomyces*, and *Pseudomonas* are more abundant microbes found in the plant rhizosphere.^68^

Ecological balance is sustained by the microbial network by both positive interaction (for example, mutualism and commensalism) and negative interaction (such as competition and predation).^69^ Microbial communities play a key role in shaping plant performance and soil functioning.^70^ One of the earliest studies on the effect of sound on bacterial communities has already been conducted.^55^ Results have shown that sound has the potential to enhance both the effectiveness and growth rate of microbes. The connection between sound and microbial activity is evident and deserves focused attention from researchers.

Notably, root diseases caused by pathogenic microorganisms such as *Rhizoctonia solani* can be suppressed through beneficial microbial community interactions, for instance, through the combined presence of *Chitinophaga* and *Flavobacterium.*^39^ In the rhizosphere, such interactions play a crucial role in plant health, as beneficial consortia can limit pathogen proliferation while simultaneously promoting nutrient availability and plant growth. These microbial networks, often involving genera such as *Pseudomonas*, *Lysobacter*, and *Bacillus,*^29^ contribute to improved plant performance by enhancing nutrient cycling and stimulating plant defense responses.^71^ Given the sensitivity of microbial communities to environmental stimuli or cues, further in-depth research is needed to understand how sound vibrations may influence the rhizospheric microbial co-occurrence and, consequently, their functional roles in plant-microbe interactions.^32^

Sounds of different frequencies, as mechanical vibrations, can act as signaling cues in both interkingdom and intrakingdom communication among microbes, likely through mechanosensitive pathways.^72‐75^ Matsuhashi et al. conducted an experiment to investigate the impact of sound on microbial communication. These early studies opened a new avenue of research, making it clear that microbial populations can be regulated through sound vibrations. This discovery holds significant implications, whether for industrially important microbes or those contributing to ecological communities.^75^ A summary of various studies on how sound impacts microbial species is presented in Table 2.

**Table 2.** Describing the impact of sound vibrations of different frequencies on the microbial community.

Species	Type of sound treatment	Type of article	Major findings	References
Gut microbial community of Mice	- Sound frequency 20–20,000 Hz - Classical music - Whole growing season	Experimental paper	- Increase of Firmicutes/Bacteroidetes ratio - Increase in the diversity of genus *Candidatus Jettenia*, *Denitratisoma*, and SM1A02	[76]
Phyllospheric microbiota of *Vitis vinifera* L. (cultivar “Syrah)		Experimental paper	- Increased growth of *Bacillus, Kocuriam,* and *Sphingomonas* - Enhanced abundances of taxa of *Methylobacterium*, *Sphingomonas*, *Bacillus,* and *Sporobolomyces roseus*	[77]
*E. coli* K-12	1) 250–16,000 Hz sound, frequency, 80 dB sound intensity, and 55 dB sound power level 2) 0–100 dB sound intensity 55 dB sound power level 3) 8 KHz frequency, 80 dB sound intensity and 55–63 dB sound power level	Experimental paper	- *E. coli* K-12 cells are elongated. - Increase in the microbial biomass.	[78]
Green microalgae *Dunaliella salina*	- Sound frequency 100, 200, 500, and 1000 Hz -Sound intensity 90 dB - 15 d - 18 d culture	Experimental paper	- Microalgae treated with the 200 Hz intensity sounds showed increased growth - More tolerance to abiotic stress	[79]
Yeast *Saccharomyces cerevisiae*	- Audible range (20 Hz to 20 kHz),	Experimental paper	- Growth rate increased 23 - Showed significant differences in the volatilomes - Upregulated citrus-related aroma compounds	[80]
Yeast *Saccharomyces cerevisiae*	- Audible range 800–2000 Hz	Experimental paper	- Decreasing beer fermentation time without significantly influencing flavor	[81]
plant growth-promoting fungus *Trichoderma harzianum*	- 80 dB sound pressure level (SPL) at a peak frequency of 8 kHz and a bandwidth at −10 dB from the peak of 6819 Hz	Experimental paper	- Increased fungal biomass and enhanced *T. harzianum* conidia (spore) activity	[82]
*Pseudomonas aeruginosa*	- Sound frequency 100 Hz - 48 h culture.	Experimental Paper	- Fatty acids Increased *- fabY*, *fade*, and *pqsA* genes altered	[83]
Eight microbial cultures	- Sound frequency 41 to 645 Hz - Sound intensity 95–110 dB - Music	Experimental paper	- Increased growth of *Xanthomonas campestris*, *Chromobacterium violaceum*, *Serratia marcescens*, *Staphylococcus aureus*, *Streptococcus pyogenes*, *Streptococcus mutans*, *Saccharomyces cerevisiae*, and *Candida* *albicans* except *S. marcescens*	[84]
Bacterial cells	38–689 Hz	Experimental paper	- Increased antibiotic susceptibility (3.81%–18.69%), accelerated metabolism, and altered membrane permeability. It also significantly changed intracellular protein content and cation (calcium/potassium) concentrations	[85]

Note: direct comparison among studies is limited by substantial differences in sound intensity, exposure duration, and experimental setup.

These findings suggest that sound vibrations may influence plant health not only through direct effects on microbial communities but also by modulating plant physiological responses, thereby shaping the complex interactions at the plant–microbe interface. Indeed, sound vibration acts as a dual-purpose tool in agriculture: while specific frequencies stimulate beneficial microbes, others can be targeted to inhibit or destroy harmful pathogens by activating the defense genes of the plants against pathogens. For example, an experiment on *Arabidopsis thaliana* showed that sound vibration priming activated defense-related genes against the pathogen *Botrytis cinerea*. The results demonstrated that plants treated with sound vibrations had a significantly higher tolerance to the infection compared to the untreated control group.^61^ Another study demonstrated that *Arabidopsis thaliana* exhibited improved tolerance to the biotic stress caused by *Ralstonia solanacearum* when exposed to sound vibrations. Treated plants showed modifications in the promoter regions of cytokinin and glucosinolate biosynthesis signaling genes, leading to enhanced immunity. A downregulation of *miR397b* expression was observed, which contributed to improved cell wall reinforcement through lignin accumulation. This was facilitated by the activation of three *LACCASE* transcripts when treated with 10 kHz sound vibrations. In summary, sound vibrations enhanced plant immunity against *R. solanacearum* by stimulating epigenetic modifications in genes involved in secondary metabolite biosynthesis.^86^

Studies have already demonstrated that both plants and microbial communities are independently responsive to acoustic stimuli. However, the specific impact of sound on the rhizosphere, the critical interface where these two meet, remains a significant research gap. While it can be hypothesized that sound vibrations could positively modulate the rhizospheric microbiome to enhance plant health, empirical evidence directly analyzing this interaction is still lacking. It can also be proposed that acoustic vibrations act as a primary trigger, potentially coordinating a joint response where direct microbial perception works alongside plant-mediated physiological changes to shape the soil microbiome. Further research is needed to analyze how sound-treated rhizospheric communities specifically evolve and how this might improve plant productivity.

## Sound vibrations and root dynamics

Plants respond to environmental changes by enhancing their organs, with roots being vital for absorbing essential nutrients and water to ensure optimal growth and development. Strengthening the root system can greatly enhance plant growth, boost resilience to biotic and abiotic stresses, and improve both product quality and yield by optimizing nutrient and water uptake.^87^^,^^88^ Consequently, numerous studies have concentrated on improving crop growth performance by encouraging root development and expansion.^89^^,^^90^

While the effects of sound on animals have been extensively documented, its influence on plants remains comparatively less explored. Although research in plant bioacoustics is growing, the vast majority of studies focus on shoots, gene expression, and overall biomass. Consequently, there is a significant lack of data regarding the impact of acoustic stimuli on the root system. The following sections discuss the existing findings while highlighting this critical research gap.

Experimental studies have revealed that sound waves have a notable effect on seedling roots, as evidenced by the elongation of hypocotyls in *Arabidopsis* seedlings exposed to sound treatment.^91^ Additionally, sound waves have been found to enhance growth by boosting the levels of growth-promoting hormones such as indole acetic acid (IAA), salicylic acid (SA), and jasmonic acid (JA) in *Arabidopsis.* Furthermore, exposure to 100 Hz and 100 Hz + 9 kHz sound frequencies positively influences early root development in *Arabidopsis*. Moreover, sound treatment promotes root growth, ultimately boosting plant productivity by increasing cytokinin levels while reducing auxin concentrations.^31^

The response of a plant's shoot system to sound waves differs from that of the root system. Roots exhibit a highly frequency-specific reaction to sound stimuli. For instance, in *Zea mays*, root tips have been observed to move toward the source of sound waves.^92^ Interestingly, roots showed varying responses to different sound frequencies, with the most significant reaction occurring within the 200–300 Hz range.^92^

Some of the roots showed behavioral response to the sound for example roots of *Pisum sativum* can find the water even in the absence of moisture by perceiving the sound vibrations produced by the water.^64^ Interestingly, findings revealed that plants prefer to move toward the moisture instead of the sound vibrations produced by the water. It suggests that plants may first perceive the sound of water, and then correctly detect the actual moisture presence. Moreover, the authors played the water sound in the speaker, but the plant moved away from the speaker. At this moment the authors hypothesized that maybe plants move away from the water sound due to any other clue, just like the magnet in the speaker.^64^

Recent research has confirmed that 10 kHz sound vibrations serve as a potent physical trigger, eliciting induced resistance in *Arabidopsis* roots against *R. solanacearum*. Notably, these acoustic stimuli induce transcriptional changes that are distinct from those triggered by traditional chemical elicitors, highlighting a unique regulatory pathway for plant defense^28^ The interaction of plant roots, microbial communities, and sound is a promising research field. Researchers from different fields, like advanced imaging technology, environmental sciences, and food safety technology, can explore more facts about the impact of the sounds on the plant and specifically on the plant root system. This is a promising approach to innovative agricultural practices that enhance plant growth, development, and stress tolerance. However, the precise physiological and molecular mechanisms by which sound waves impact specific roots and shoot tissues are not yet fully understood.

## Sound vibrations and biotic interactions in the plant holobiont

The plant holobiont, which refers to the complex assemblage of a host plant and its diverse microbial associations, functions as a single biological unit where members influence each other's growth and overall health.^93^^,^^94^ This microbiota plays a crucial role throughout the plant's life cycle, from germination to yield,^95^^,^^96^ and maintains a healthy balance through interactions between pathogens and antagonistic microbes.^97‐99^ These interactions are traditionally viewed as chemical. Sound vibrations are now recognized as a vital physical stimulus that can modulate this entire system.^100^^,^^101^

Acoustic stimuli, whether natural, anthropogenic, or even microbially generated, serve as environmental triggers within the holobiont. It can be hypothesized that microbial metabolic activity may generate specific vibrational signatures, that can influence microbial community dynamics and, consequently, affect the entire plant holobiont, including plant physiology. Accordingly, it has been found that plants perceive sound by activating mechanoreceptors and signal transduction pathways,^101^^,^^102^ leading to increased resistance against pathogens like *Botrytis cinerea.*^61^ Crucially, the microbial members of the holobiont are also responsive, microbiota exposed to sound show enhanced metabolism, antibiotic production, and stress tolerance.^78^^,^^84^^,^^103^ The rhizospheric beneficial microorganisms establish symbiotic relationships with plants, significantly enhancing their resilience against both biotic and abiotic stressors.

The plant holobiont extends beyond microbial communities to include interactions with insects. Many evidences also shows that the holobiont utilizes acoustic cues for ecological survival; plants can perceive the vibrations of chewing larvae or the buzzing of bees to trigger defense or pollination responses.^15^^,^^104^^,^^105^ Plants are able to use acoustic reflectors to guide bats for pollination.^104^

Integrating plant holobiont interactions with acoustic stimuli could be a foundational strategy for advancing sustainable agriculture amidst global environmental challenges. By leveraging sound vibrations to modulate these biological interactions, we can unlock new pathways for enhancing crop resilience and productivity, positioning bioacoustics as a transformative tool for the future of sustainable agriculture. These findings suggest that sound may act as a cross-kingdom communication tool, maintaining the health and functional balance of the plant holobiont.

## Conceptual physiological hypotheses linking sound vibrations to plant–microbe interactions in the rhizosphere

Despite the growing number of studies reporting sound- or vibration-induced effects on plant growth, development, and associated microbial communities, a coherent physiological framework linking acoustic stimuli to functional plant traits and rhizospheric processes is still lacking.^13^^,^^14^ To move beyond a descriptive overview, we propose a set of testable, physiology-driven hypotheses that integrate current evidence on plant mechanosensing, root biology, and plant–microbe interactions within a holobiont perspective.

Firstly, sound and vibration stimuli are hypothesized to act as mechanical cues rather than classical sensory signals. These cues may be perceived by plant tissues through mechanosensitive cellular components, including the cell wall, plasma membrane continuum, the cytoskeleton, and potentially mechanosensitive ion channels such as the MSL, PIEZO, OSCA, MCA, GLR, and CNGC families.^46^^,^^47^^,^^50^^,^^54^^,^^106^ It is proposed that mechanical perturbations induced by sound waves generate membrane tension and cell wall deformation, which could lead to rapid ion fluxes, particularly Ca^2+^, and the activation of downstream signaling cascades.^16^^,^^39^^,^^55^^,^^62^^,^^63^ These early events are thought to constitute a primary physiological perception step linking physical acoustic energy to biological responses, although the exact molecular pathways remain to be fully elucidated.

Secondly, following mechanosensory perception, sound-induced Ca^2+^ waves interact with hormonal signaling networks, including auxin, cytokinin, jasmonic acid, salicylic acid, and abscisic acid pathways.^31^^,^^33^^,^^34^^,^^39^ This interaction is proposed to alter root meristem activity, cell elongation, and lateral root formation in a frequency- and intensity-dependent manner.^49^^,^^50^^,^^64^ Changes in root system architecture—such as increased root length, branching, or root hair development—represent key functional traits that directly influence water and nutrient uptake efficiency and overall plant performance under both optimal and stress conditions.^50^^,^^70^^,^^89^^,^^90^^,^^99^^,^^102^

Thirdly, modifications in root growth and cellular signaling are expected to be accompanied by quantitative and qualitative changes in root exudation.^19^^,^^45^ Sound-induced shifts in primary and secondary metabolism may affect the release of sugars, amino acids, organic acids, phenolics, and signaling molecules into the rhizosphere. These compounds play a central role in shaping microbial recruitment and activity.^17^^,^^23^ Thus, sound vibrations are hypothesized to indirectly modulate the chemical environment of the rhizosphere by altering plant-controlled exudation patterns rather than acting directly on soil microorganisms.

Fourthly, although several microbial taxa have been shown to respond to acoustic or vibrational stimuli in isolated culture conditions, evidence for direct sound perception by complex rhizospheric microbial communities remains limited.^33^^,^^34^^,^^36^^,^^107^^,^^108^ We therefore hypothesize that most observed changes in rhizosphere microbiome composition and function under sound exposure arise from plant-mediated effects, including altered root architecture, exudation profiles, and immune signaling.^21^^,^^22^^,^^29^^,^^32^^,^^109^ These plant-driven changes may selectively favor beneficial microbial taxa, such as plant growth–promoting rhizobacteria or antagonists of soil-borne pathogens, ultimately contributing to improved nutrient availability and stress tolerance.

Moreover, at the holobiont level, the integration of mechanotransduction, root physiological responses, and microbial community shifts is expected to result in emergent agronomic traits, including enhanced growth, improved water-use efficiency, increased nutrient uptake, and greater resilience to biotic and abiotic stresses.^12^^,^^47^^,^^49^^,^^50^^,^^53^^,^^89^ These traits provide a functional link between sound stimulation and horticultural or agronomic performance, supporting the potential application of sound-based approaches as low-impact tools in sustainable agriculture (Table 3).

**Table 3.** Conceptual synthesis of the proposed physiological pathways linking sound-induced mechanotransduction to root traits, rhizospheric processes, and agronomic outcomes within a plant holobiont framework.

Sound-induced stimulus	Primary physiological process	Root-level response	Rhizospheric consequence	Agronomic/horticultural trait	References
Mechanical vibration (airborne or substrate-borne)	Activation of mechanosensitive ion channels (MSL, PIEZO, OSCA, MCA, GLR, CNGC) and Ca^2+^ influx	Enhanced root meristem activity; altered cell elongation	Indirect modulation of microbial recruitment via root growth patterns	Increased root vigor and soil exploration capacity	[107,110]
Frequency-specific sound exposure	Ca^2+^-dependent signaling and cytoskeletal reorganization	Changes in root architecture (root length, branching, root hair density)	Expansion of the rhizospheric niche and microbial colonization surface	Improved nutrient uptake efficiency (N, P, micronutrients)	[45,100]
Sound-induced mechanotransduction	Hormonal rebalancing (IAA, cytokinins, JA, SA, ABA)	Optimized root-to-shoot signaling and resource allocation	Selective enrichment of plant growth–promoting rhizobacteria (PGPR)	Enhanced plant growth and biomass accumulation	[109]
Sustained sound stimulation	Modulation of primary and secondary metabolism	Quantitative and qualitative changes in root exudation	Shifts in microbial community composition and functional potential	Increased nutrient availability and rhizosphere functionality	[28,101]
Sound-triggered stress signaling	ROS–Ca^2+^ crosstalk and antioxidant activation	Root acclimation to mechanical and abiotic stress	Promotion of stress-adapted and antagonistic microbial taxa	Enhanced tolerance to abiotic stress (e.g. drought, salinity)	[40,111]
Sound-induced immune priming	Activation of ISR/SAR-related pathways	Reinforced root cell walls and altered defense metabolism	Suppression of soil-borne pathogens through beneficial microbes	Improved resistance to biotic stress and reduced disease incidence	[28,112]
Integrated sound–root–microbe interaction	Holobiont-level coordination of signaling pathways	More resilient and plastic root system	Stabilized and functionally redundant microbial networks	Increased yield stability and resilience under variable environments	[104]

**Figure 2.**
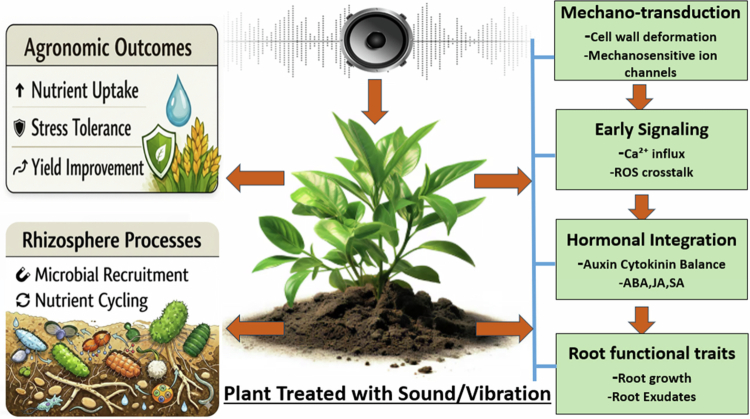
Physiological model linking sound vibrations to root function and rhizospheric processes within the plant holobiont. Sound-induced mechanical stimuli are perceived by plant tissues through mechanosensitive structures at the cell wall–plasma membrane interface, leading to the activation of mechanosensitive ion channels and Ca^2+^ influx. Calcium-dependent signaling interacts with hormonal pathways (auxin, cytokinins, ABA, JA, SA), modulating root meristem activity, root architecture, and metabolic status. These root-level physiological changes alter the quantity and composition of root exudates, indirectly reshaping rhizospheric microbial community assembly and function. The integration of these processes at the plant–soil interface results in emergent agronomic traits, including enhanced nutrient acquisition, stress tolerance, and plant performance.

The conceptual framework developed in this review is summarized in Figure 2, which depicts the physiological pathways through which sound vibrations may influence plant–soil interactions. By explicitly linking mechanosensory perception, calcium and hormonal signaling, root functional traits, and rhizospheric microbial responses, the figure provides a mechanistic interpretation of the experimental evidence discussed in the text and highlights testable links between sound stimulation and agronomic outcomes.

## Integrated experimental platforms for studying acoustic signaling in the plant holobiont

Understanding how plants and microbes perceive and respond to sound requires integrating tools from acoustics, molecular biology, plant physiology, and microbiome science. Several advanced and emerging techniques are now enabling researchers to explore these interactions with much greater precision. Precise sound exposure systems are essential for controlled experiments. Modern setups include programmable speakers, vibration platforms, and frequency-specific sound generators, which allow accurate manipulation of sound intensity, duration, and frequency.^28^^,^^103^ These systems have been widely used to study plant sound responses in *Arabidopsis*, rice, ornamental species, and microbial cultures.^15^^,^^28^^,^^103^ Ecoacoustic tools such as directional microphones, accelerometers, and laser doppler vibrometers enable non-invasive detection of plant- or microbe-produced sounds.^113^ These instruments can quantify vibrations or airborne ultrasounds emitted during water stress, nutrient limitation, or microbial metabolism. Sound often triggers mechanical and calcium-based signaling. Advanced microscopy tools are increasingly used to visualize these early events.

Confocal and fluorescence microscopy can track calcium fluxes and cytoskeletal changes during sound perception.^114^^,^^115^ Atomic force microscopy (AFM) and nanoindentation are employed to measure cell wall mechanics, stiffness, and mechanotransduction under mechanical or acoustic stimulation.^116^

High-speed video microscopy enables analysis of sound-induced trichome or root movements and other rapid mechanical responses.^116^ Together, these tools may help to identify the mechanical steps linking sound perception to cellular responses. Mechanosensitive channels such as MSL, OSCA, MCA, GLR, CNGC, and PIEZO play essential roles in mechano-transduction and are likely involved in sound perception. Their functions are commonly studied using electrophysiology (i.e. patch-clamp recordings),^28^ as well as mutant analyses and heterologous expression systems.

Extensive studies on these channels in *Arabidopsis* provide mechanistic foundations for sound sensing at membranes and cell walls.^113^^,^^117^^,^^118^ Recent work further highlights mechanosensitive channels as central components of plant mechanotransduction and adaptive responses to mechanical cues. Multi-Omics Tools for Sound-Induced Responses Sound triggers transcriptomic, proteomic, metabolomic, and hormonal changes in plants and microbes. Modern multi-omics methods allow comprehensive profiling of these responses.^119^^,^^120^

Since microbes play a crucial role in mediating or responding to sound, modern microbiome tools are essential. Amplicon sequencing (16S/ITS) is widely used to characterize shifts in root and soil microbiomes in response to environmental cues and experimental treatments.^108^^,^^120^ Metagenomics and meta-transcriptomics provide deeper insight into functional gene repertoires and active processes in plant-associated microbial communities. Root exudate profiling links sound-induced changes in plant metabolite release with microbial recruitment and activity in the rhizosphere.^108^ Evidence from plant–microbe interaction studies indicates that environmental signals can alter microbial activity, promote beneficial taxa, and reshape plant–microbe interactions, suggesting similar potential for sound-mediated effects.

**Figure 3.**
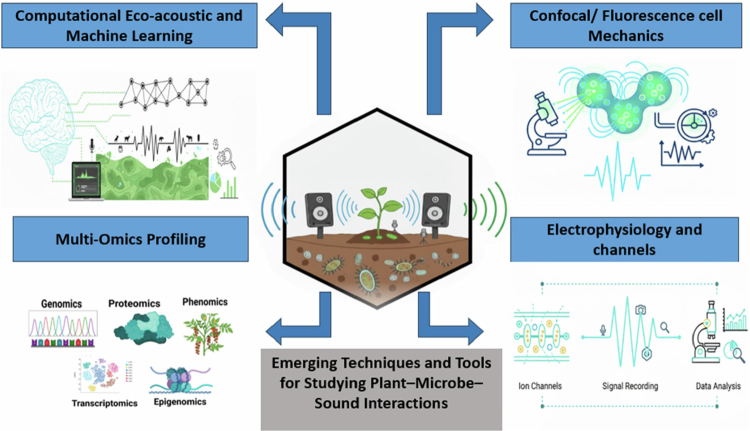
Graphical representation of emerging techniques and tools used to study plant–microbe–sound interactions, including computational eco-acoustics and machine learning, multi-omics approaches, confocal microscopy, and electrophysiological analysis of ion channel alterations. These methods enable the detection of sound-induced effects on different plant species by comparison with control groups.

The physiological framework outlined above provides the basis for identifying appropriate experimental approaches to investigate sound-mediated plant–microbe interactions, as summarized in Figure 3.

Ultrasound and low-frequency sound platforms allow simulation of mechanical stress under controlled conditions. These tools are used to investigate: oxidative stress, antioxidant responses, membrane integrity, and hormone modulation in plant tissues.^121^^,^^122^ Such systems help map similarities and differences between sound-induced stress and other abiotic stresses, such as drought, salinity, or mechanical perturbation. Recent advances in computational ecoacoustics enable: classification of sound signatures emitted by plants and microbes, and prediction of stress states or environmental conditions from acoustic profiles alone.^123^ These computational and machine-learning applications hold promises for decoding sound-based communication and stress signaling within the plant holobiont.

## Experimental limitations and reproducibility issues in plant holobiont bioacoustics

Current research on plant–microbe–sound interactions has several methodological challenges that make the results difficult to interpret. One major problem is distinguishing the true effects of airborne sound from other factors, such as vibrations traveling through the soil, air movement, or electromagnetic fields produced by speakers and electronic devices. Many experimental setups use loudspeakers or vibration systems that generate multiple types of stimuli at the same time, making it hard to confirm whether the observed plant or microbial responses are caused specifically by sound. In addition, some studies use single-frequency tones or music-like sounds at high or unnatural intensities. These conditions may not reflect sounds that plants experience in natural environments, and the observed responses could simply be stress reactions or responses to mechanical disturbance rather than meaningful biological signals.

Experimental designs often lack detailed control and reporting of sound properties, such as sound pressure levels at the plant surface, frequency ranges, and how sound is distributed in space. Small differences in distance, orientation, or chamber design can strongly change the sound experienced by the plant, leading to inconsistent or non-reproducible results between laboratories.

From a biological perspective, many studies focus on short-term experiments or measure only a single outcome. This provides a limited understanding of how sound affects plant–microbe systems over time. The use of time-resolved approaches, such as multi-omics and microbiome analysis, is still limited, making it difficult to distinguish short-lived responses from long-term effects. In many cases, microbial involvement is assumed rather than directly measured, as few studies simultaneously analyze plant physiology, root exudates, and microbial community changes under controlled sound treatments. As a result, it remains unclear whether sound affects plants directly or indirectly through changes in microbial activity.

Overall, reproducibility and standardization remain major issues in plant bioacoustics and soil eco-acoustics. Differences in equipment, calibration methods, data processing, and statistical analyses can lead to very different conclusions, even when similar sound treatments are applied. For this reason, researchers are increasingly calling for standardized protocols, detailed reporting of acoustic setups, and validation across multiple laboratories. Addressing these limitations is essential to reliably identify real sound-mediated effects and to build a solid framework for studying plant–microbe–sound interactions.

## The significance of sound responsiveness in the plant holobiont for agricultural applications

Our understanding of plant responses to environmental stimuli is evolving to recognize their intricate interactions with the surrounding soil and microbial communities. The ability of plants, as part of a larger holobiont, to perceive and react to sound holds significant implications for agricultural practices. Moving beyond a plant-centric view to embrace the holobiont is crucial for developing effective and sustainable sound-based agricultural applications.

As reported in this review, studies have indicated that sound waves can influence various aspects of plant growth and physiology.^28^ Sound also acts as a signaling cue that influences plant behavior by altering gene expression. This modification, in turn, activates various biochemical activities within the plant.^108^ Moreover, sound vibrations also affect plant microstructures, including porosity, intercellular spaces, and cellular organelles. Sound application can create or modify microchannels within plant cells and may alter the size and structure of intercellular spaces.^124^ Furthermore, the application of sound waves has also been shown to positively influence crop yield.^38^ Indeed, agri-wave technology, involving acoustic wave impulses, has demonstrated a significant enhancement in crops of agronomic interest, such as growth^13^ accelerated ripening,^125^ boosted production,^13^ and increased overall quality.^32^

Interestingly, while direct research on the impact of sound on the entire microbial community within these sources is limited, it is reasonable to infer that vibrations propagating through the soil matrix could influence microbial activity and interactions. The rhizosphere is a critical interface where plant roots and soil microorganisms engage in complex exchanges that are vital for plant health.^126^ Plant growth-promoting rhizobacteria (PGPR), such as *Bacillus* spp. and *Pseudomonas* spp., are widely utilized in the agricultural sector to enhance plant health.^127^ These beneficial microbes support plant growth by fixing atmospheric nitrogen and increasing the availability of phosphorus and other essential minerals through solubilization processes.^128^^,^^129^

Changes in the physical environment, such as vibrations, could potentially affect these interactions. It is also worth considering the potential role of root exudates; they play a crucial role.^130^ If sound influences root exudation,^64^ For example, in *Arabidopsis* plants, sound stimulation has been shown to enhance root meristem activity and promote root growth by increasing the concentrations of cytokinin and auxin.^31^ It could indirectly impact the microbial community. The importance of the microbiome of the plant holobiont for plant health and stress resistance is increasingly recognized.^131^ Understanding how sound influences this component is essential for holistic agricultural applications.

**Figure 4.**
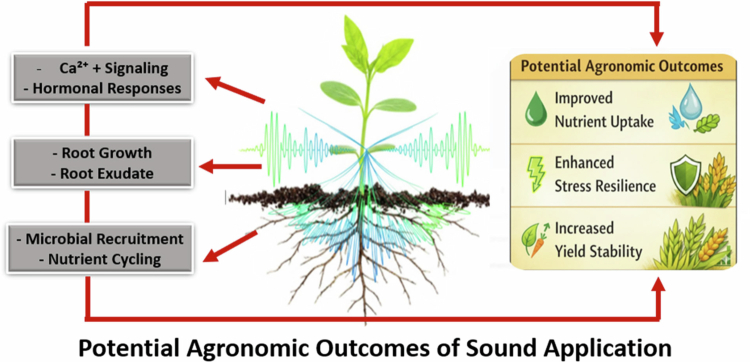
Acoustic stimulation induces mechanotransduction and root-rhizosphere interactions, facilitating microbial recruitment. These processes collectively result in enhanced crop performance, specifically improved nutrient acquisition and stress tolerance.

Considering the interconnectedness of the plant, soil, and microorganisms is paramount for sustainable agriculture.^132^ Accordingly, agricultural applications of sound technology should adopt this holobiont perspective to maximize benefits. Modulating sound environments could potentially be used to foster beneficial soil microbes, enhance nutrient uptake, and improve plant resilience to both biotic and abiotic stresses, thus reducing the reliance on chemical inputs. Figure 4 illustrates a conceptual scheme suggesting that acoustic stimuli could improve root-microbe interactions and microbial recruitment, potentially leading to better nutrient uptake and overall crop performance.

Literature is full of studies on the impact of sounds on the plants as described in Table 1. Microbial community has also been explored many times as the reference of the sound waves. But none of the study is reported yet that explore the impact of the sound waves on the plant microbial community.

## Conclusion and future perspectives

Growing evidence indicates that sound and vibration stimuli can influence plant growth and stress responses; however, their integration into plant–soil systems has remained largely descriptive. By adopting a plant holobiont perspective, this review provides a physiological framework in which sound vibrations are interpreted as mechanical cues perceived by plant tissues through mechanotransduction pathways. These early signaling events, involving calcium and hormone-mediated responses, are proposed to modulate root functional traits, root exudation, and downstream rhizospheric microbial processes.

Importantly, the framework developed here suggests that most reported changes in rhizospheric microbial communities under sound exposure are likely indirect and plant-mediated, rather than the result of direct microbial sound perception. This interpretation reconciles disparate findings in the literature and highlights root physiology as a central hub linking acoustic stimuli to plant–microbe interactions. By explicitly connecting sound perception to root traits, rhizospheric dynamics, and agronomic outcomes, this review moves beyond phenomenological observations and identifies testable hypotheses for future research. Integrating eco-acoustics, plant physiology, and microbiome science may enable the development of sound-based approaches as low-impact tools to enhance nutrient acquisition, stress resilience, and sustainability in agricultural systems.

The potential for plants and the soil microbiome to be sensitive to sound presents a fertile ground for developing novel agricultural technologies. Both research in the future and practical applications must embrace the concept of the plant holobiont, recognizing the multifaceted interactions between the plant, the soil environment, and its microbial inhabitants.

To conclude, the integration of eco-acoustics, not only for soil health monitoring but also for actively shaping biological response, has the potential to significantly enhance our ability to manage these complex systems. Emerging technologies such as agri-wave platforms, low-frequency vibration treatments, and bio-acoustic signaling devices hold promise as worthy allies in the quest to improve plant growth, stress tolerance, and beneficial microbial populations while reducing chemical inputs.

**Figure 5.**
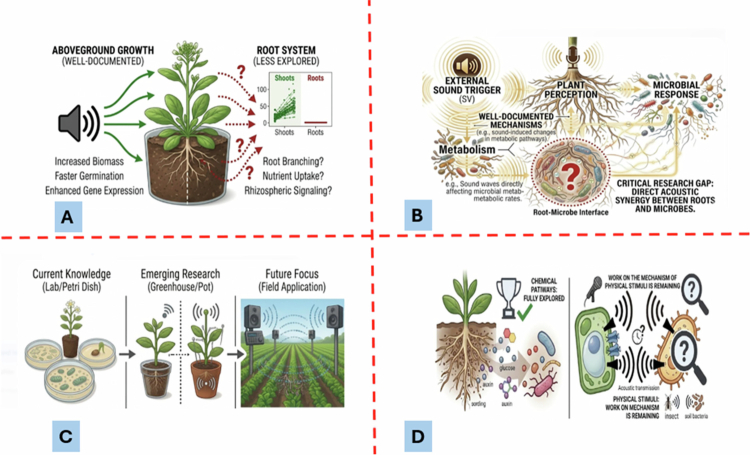
Illustrates the multidimensional landscape of plant-microbe acoustic communication, starting with Panel (A), which contrasts well-documented aboveground growth responses, such as increased biomass and faster germination with the “less explored” subterranean root system, highlighting significant gaps in our understanding of root branching and rhizospheric signaling. Panel (B) maps the acoustic communication pathway from external sound triggers to plant perception and microbial response, specifically identifying the root-microbe interface as a critical research gap where direct acoustic synergy remains poorly understood. This transition of research scales is further detailed in Panel (C), which outlines the progression from current in-vitro and lab-based knowledge to emerging greenhouse studies, ultimately targeting a future focus on large-scale field applications for sustainable yield enhancement. Finally, Panel (D) provides a comparative analysis of signaling mechanisms, showing that while chemical pathways involving auxin and glucose diffusion are already fully explored, the underlying physical mechanisms and mechanotransduction of acoustic stimuli represent the primary frontier where work is still remaining.

By tuning in to the subtle acoustic languages of plants and microbes, new possibilities for sustainable agriculture are unveiled, where sound is employed as a gentle, natural way of affecting the health and productivity of entire ecosystems. This non-invasive and holistic approach is a tangible step in the direction of climate-resilient, ecologically aligned farming systems. Despite the established literature, several key questions remain unanswered (Figure 5).

Looking ahead, the field of plant bioacoustics is poised to evolve through several critical research frontiers that will reshape our understanding of botanical communication. Future studies sprioritize the investigation of mechanosensitive channels to decode the precise physiological mechanisms governing how plants perceive auditory stimuli. While research has historically emphasized the shoot system and fruit characteristics, a vital shift toward exploring the impact of sound on root systems and rhizospheric microbial communities is necessary to understand the holistic health of the plant. Moreover, the study of plant sound emissions promises to usher in a transformative era of plant communication research, potentially revealing acoustic signaling as a fundamental tool for environmental interaction. To ultimately integrate these insights into sustainable agriculture, the field must transition from controlled laboratory settings to extensive field trials, bridging the gap between theoretical discovery and the practical application of acoustic technology in large-scale crop management.

## Data Availability

Data sharing is not applicable to this article as no new datasets were generated or analyzed during the current study. All information discussed is derived from the cited literature.

## Clinical trial number in the manuscript

Clinical trial number: not applicable.
